# Measurement of the Proton and Oxide‐Ion Conductivities of Dual‐Ion Conductors by Switching the Current Direction

**DOI:** 10.1002/smtd.202500166

**Published:** 2025-05-19

**Authors:** Xiangcheng Liu, Qiuning Li, Lingping Zeng, Xiaoliang Zhou, Dehua Dong, Zongping Shao, Huanting Wang

**Affiliations:** ^1^ Department of Chemical and Biological Engineering Monash University Clayton VIC 3800 Australia; ^2^ College of Chemistry and Chemical Engineering Southwest Petroleum University Chengdu 610500 P. R. China; ^3^ WA School of Mines: Minerals Energy and Chemical Engineering Curtin University Perth WA 6102 Australia

**Keywords:** dual‐ion conductor, H^+^ conductivity, measurement, O^2−^ conductivity

## Abstract

H^+^/O^2−^ dual‐ion conductors have demonstrated superior performance in fuel cells and electrolysis cells. However, a simple and precise method for measuring the H^+^ and O^2−^ conductivities of dual‐ion conductors is lacking. This study developed electrochemical impedance spectroscopy (EIS) tests under direct current. Coupled with water electrolysis on one electrode by introducing water vapor, EIS tests can measure the individual conductivities of H^+^ and O^2−^ simply by switching the current direction. In addition, the H^+^/O^2−^ dual‐ion conductivity is measured when water vapor is applied to both electrodes. The H^+^, O^2−^ and dual‐ion conductivities of the state‐of‐the‐art BaCe_0.7_Zr_0.1_Y_0.1_Yb_0.1_O_3_‐_δ_ (BZCYYb) are measured and compared with those of other dual‐ion conductors for the first time. La_0.9_Sr_0.1_Ga_0.8_Mg_0.2_O_3‐δ_ shows H^+^, O^2−^ and dual‐ion conductivities comparable to those of BZCYYb at temperatures below 625 °C. Therefore, this study has developed a novel method to measure the ionic conductivities of dual‐ion conductors simply and precisely.

## Graphical abstract

Coupling with water electrolysis on one electrode, electrochemical impedance spectroscopy can accurately measure the individual protonic and oxygen ionic conductivities of dual‐ion conductors under a direct current, which is achieved simply by switching the current direction. The new method provides a powerful tool to develop dual‐ion conductors for various applications.

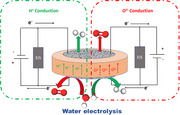

## 1 Introduction

Ionic conductors, particularly H^+^ and O^2−^ conductors, have been widely applied in fuel cells, electrolysis cells, gas sensors, gas separation membranes, and membrane reactors. Fluorite Y‐doped zirconia and Gd‐doped ceria (GDC) are typical O^2−^ conductors and some perovskites and mixed oxides have H^+^ and/or O^2−^ conductivity.^[^ ^1^, ^2^ ^]^ The membranes with H^+^ and O^2−^ conductivities are called dual‐ion conductors or mixed‐ionic conductors,^[^ ^3^, ^4^, ^5^, ^6^ ^]^ such as perovskite oxides, fluorite/perovskite composites, and palmierite oxides.

Fuel cells and electrolysis cells based on dual‐ion electrolytes demonstrate better electrochemical performance than those based on H^+^ or O^2−^‐conducting electrolytes. For example, water is formed on both the anode and cathode of fuel cells, which improves the H^+^ conductivity of the perovskite electrolyte and prevents fuel dilution on the anode side of O^2−^‐conducting cells.^[^ ^4^ ^]^ In electrolysis cells, water is electrolyzed simultaneously on both the anode and cathode sides to maximize hydrogen production.^[^ ^7^ ^]^ In addition, dual‐ion conductors have been widely applied in the electrodes of protonic cells and electrocatalysts,^[^ ^8^, ^9^ ^]^ where both H^+^ and O^2−^ are involved in reactions. However, there has been limited progress in the development of dual‐ion conductors because there is a lack of techniques for simply and precisely testing the individual conductivity ^[^ ^4^ ^]^, which is crucial for designing and controlling the dual‐ion conductivity to meet the various requirements of different applications.

Conventional methods to measure dual‐ion conductivities include water formation, gas permeation, the Hebb‒Wagner method and electrochemical impedance spectroscopy (EIS). Shao et al. tested the H^+^ and O^2−^ conductivities of the electrolyte membrane of a fuel cell by measuring the amount of water formed on two electrodes.^[^ ^10^ ^]^ Similarly, hydrogen and oxygen permeations through dual‐ion membranes were employed to calculate the individual conductivity. Although these techniques provide straightforward ways to measure individual conductivity, both methods have problems with the precise quantification of water and hydrogen due to water evaporation and hydrogen leakage issues, respectively.

EIS offers a simple way to test ionic conductivities with high accuracy. To separate the two ionic conductivities, two strategies have been proposed. In the Hebb‒Wagner method, a thin layer is coated on the conductors to block protons or oxygen ions.^[^ ^11^, ^12^ ^]^ The main challenges are the selection of pure O^2−^ conductors or H^+^ conductors and the elimination of reactions between layers during membrane preparation by sintering.^[^ ^13^ ^]^ Another strategy is to control the atmosphere around the conductors, and the H^+^ and O^2−^ conductivities are tested under hydrogen and oxygen atmospheres, respectively. This method has been widely used to test individual conductivity. A wet environment is a common working environment of dual‐ion cells, as water presents as either a product or a reactant. Many researchers have reported that wet environments generate greater electrical conductivity than dry environments do.^[^ ^14^ ^]^ This is because the dual‐ion conduction of H^+^ and O^2−^ occurs in the presence of water during EIS testing under an open circuit voltage (OCV). Water can be electrolyzed under potential to offer protons or oxygen ions, which was confirmed in this study. Therefore, conventional H^+^ and O^2−^ conductivities tested at the OCV and in wet hydrogen and oxygen/air are actually H^+^/O^2−^ dual‐ion conductivities. This is the reason that the conductivities tested under a wet atmosphere are greater than those tested under a dry atmosphere.

This study proposes a simple and precise method to measure the ionic conductivity with ESI under direct current and water vapor atmospheres, and the H^+^ and O^2−^ conductivities are measured individually by switching the current direction. The effects of the applied current and vapor partial pressure on the conductivity of BaCe_0.7_Zr_0.1_Y_0.1_Yb_0.1_O_3_‐_δ_ (BZCYYb) were investigated to set the testing conditions, and the BZCYYb conductivities were compared with those tested with conventional methods. The developed method was used to measure other dual‐ion conductors for comparison with BZCYYb, including the La_0.9_Sr_0.1_Ga_0.8_Mg_0.2_O_3‐δ_ (LSGM), BaCe_0.9_Gd_0.1_O_3‐δ_ (BCG) and BaCe_0.7_Zr_0.2_Y_0.1_O_3‐δ_ (BZCY)/GDC membranes.

## 2 Results and Discussions

### 2.1 Design and Evaluation of the Measurement Method

**Figure 1:**
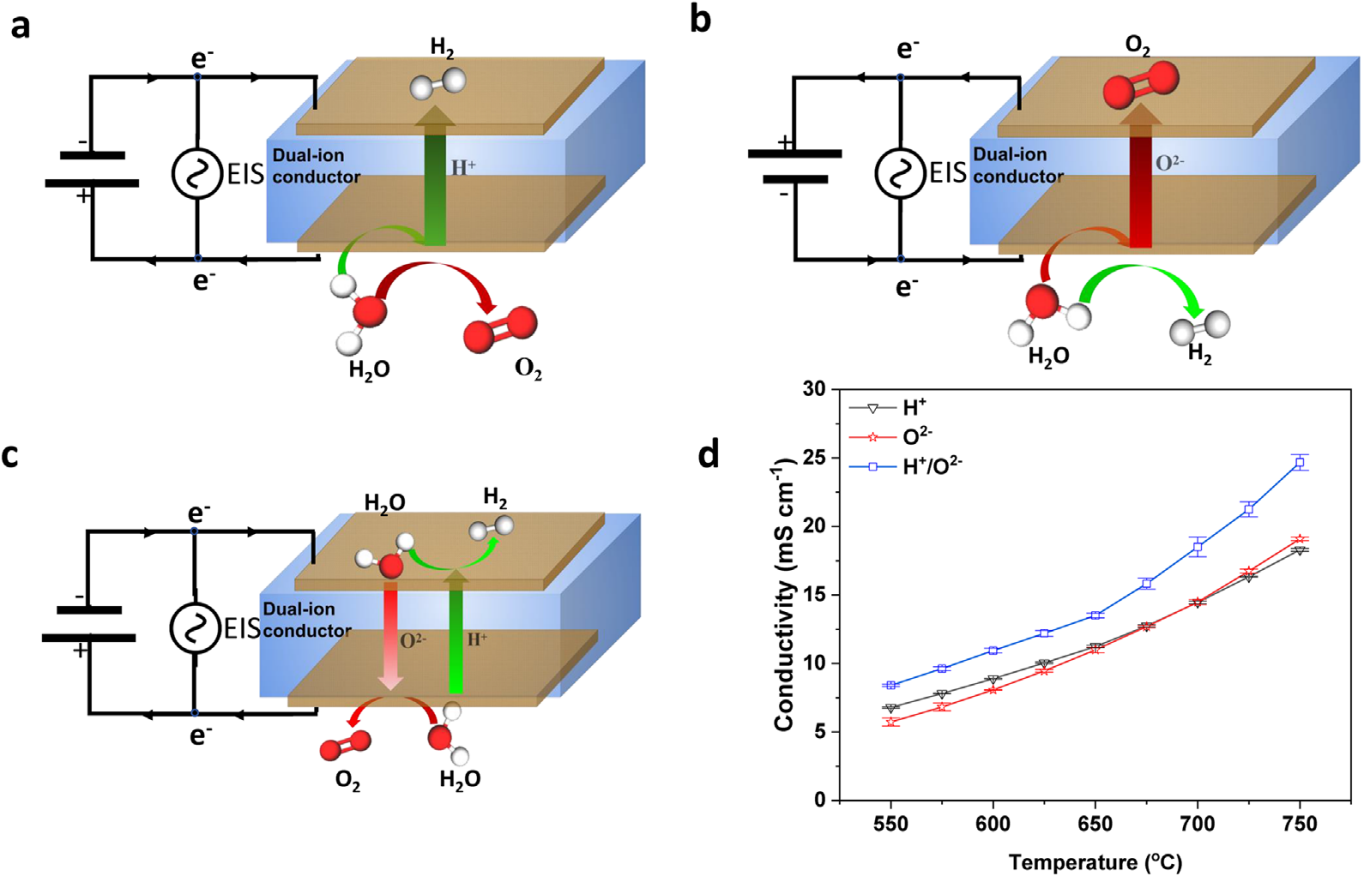
Schematic illustration of the measurements of H^+^ conductivity (a), O^2−^ conductivity (b), dual‐ion conductivity (c), and ionic conductivities (d) of the BZCYYb membranes fed 3 vol% H_2_O/Ar. The error bars represent the means ± SDs, n = 3.

Using water electrolysis to generate H^+^ or O^2−^ instead of hydrogen or oxygen in conventional tests of ionic conductivity, we propose ESI tests under a direct current, which can measure the individual conductivities of H^+^ and O^2−^ by switching the current direction. As shown in **Figure** **1**a, ESI is tested under a direct current (e.g., 10 mA), and water is electrolyzed to generate H^+^ and O_2_ on the positive electrode (H_2_O → 2H^+^ + 0.5O_2_ + 2e^−^). H^+^ conduction occurs through the membranes under potential, and H^+^ combines with H_2_ on the negative electrode (2H^+^ + 2e^−^ → H_2_). As a result, H^+^ conductivity was measured via EIS. When the current direction is switched (Figure 1b), water electrolysis generates O^2−^ and H_2_ on the negative electrode (H_2_O + 2e^−^ → O^2−^ + H_2_), and O^2−^ conduction occurs through the membranes under potential. Hence, the ESI can measure O^2−^ conductivity simply by switching the current direction. For the test of H^+^/O^2−^ dual‐ion conductivity (Figure 1c), water vapor is fed on both sides of the ionic membranes, and water electrolysis under potential generates H^+^ and O^2−^ simultaneously on both the positive and negative electrodes as above. H^+^ and O^2−^ conduction across the membranes occurs simultaneously under potential. Accordingly, the dual‐ion conductivity was measured under the same current as that used for the tests of H^+^ and O^2−^ conductivities. Therefore, H^+^, O^2−^, and dual‐ion conductivities can be readily and precisely measured via the proposed EIS tests coupled with water electrolysis under a direct current.

As a typical dual‐ion conductor, BZCYYb powder was synthesized via a solid‐state reaction at 1000 °C, and BZCYYb membranes were prepared by sintering powder pellets at 1450 °C. The density of the membranes was 6.11 g cm^−3^. The XRD patterns of the BZCYYb powder and membranes confirmed the presence of a pure perovskite phase (Figure S2, Supporting Information). The SEM image of the BZCYYb membrane shows a dense structure and a thickness of ≈620 µm. A porous Pt layer was coated on the membrane surface. Figure 1d shows the ionic conductivities of BZCYYb under 10 mA with the addition of 3 vol% H_2_O/Ar in one chamber and pure Ar in the other chamber. The O^2−^ conductivity is lower than the H^+^ conductivity at temperatures below 680 °C and becomes dominant at temperatures above 680 °C. These results are consistent with the reported results that BZCYYb is a dominant H^+^ conductor at low temperatures and an O^2−^ conductor at high temperatures.^[^ ^15^ ^]^ The dual‐ion conductivities are close to the H^+^ conductivities at low temperatures and the O^2−^ conductivities at high temperatures, indicating the mixed conduction of H^+^ and O^2−^. Therefore, the proposed EIS tests under direct current are valid for the measurement of H^+^, O^2−^ and dual‐ion conductivities.

### 2.2 Effects of Water Vapor Partial Pressure and Applied Current on Ionic Conductivity

**Figure 2:**
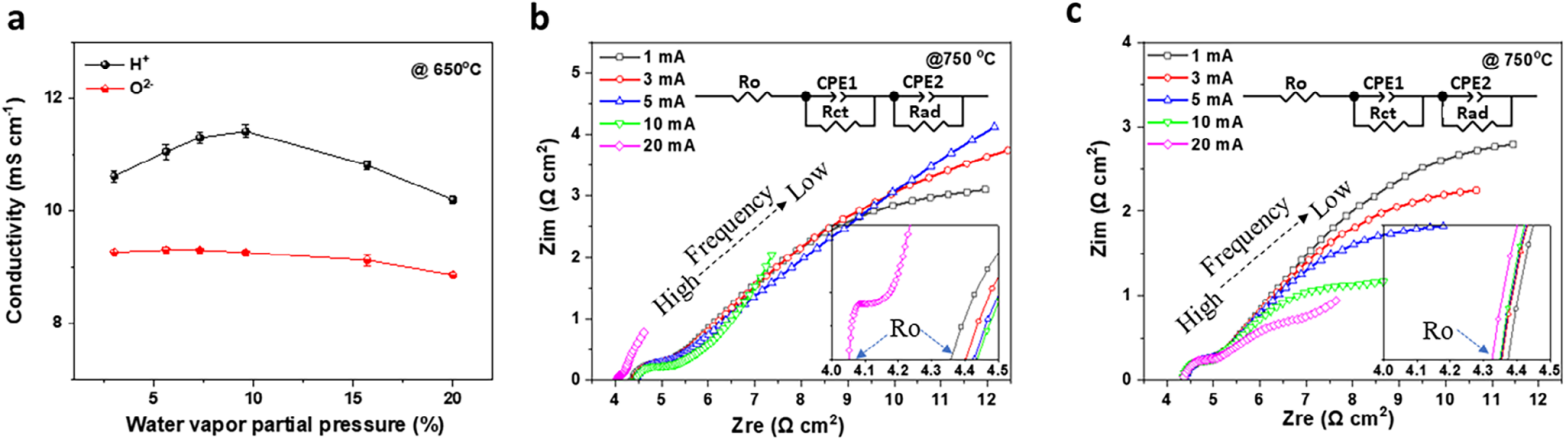
Effects of the partial pressure of water vapor (error bars represent the mean ± SD, n = 3) (a) and applied current (b and c) on the ionic conductivities: b), H^+^ conductivity; c), O^2−^ conductivity.

Water vapor is employed to provide H^+^ or O^2−^ in conductivity tests and is generally used in practical applications. It affects the ionic conductivity, especially the H^+^ conductivity. The effect of water vapor partial pressure on the conductivity was investigated by setting the water vapor pressure during the tests. As shown in **Figure** **2**a, water vapor partial pressure has a greater influence on H^+^ conductivity than on O^2−^ conductivity because water potentially participates in H^+^ conduction ^[^ ^16^ ^]^. The H^+^ conductivity increases with increasing vapor partial pressure up to 10%, and further increasing the partial pressure leads to a decrease in H^+^ conductivity, which is consistent with previous results ^[^ ^17^ ^]^. The vapor partial pressure has a similar influence on the O^2−^ conductivity at a smaller scale.

Direct current is used to select H^+^ or O^2−^ passing through membranes and applying current can alter the conductivity because Joule heating under an applied current may increase the actual temperature of the membrane and hence reduce the ohmic resistance. Accordingly, a low current is desired to minimize the current influence. To investigate the influence, the effect of the current on the conductivity was studied. As shown in Figure 2b,c, the equivalent circuit shows that the resistance at high frequencies is attributed to the ohmic resistance (R_o_) ^[^ ^18^ ^]^, which is the resistance of ionic conduction and used to calculate the ionic conductivities. The resistance at low frequencies includes the charge transfer resistance at the electrolyte/electrode interface (R_ct_) and the adsorption and/or surface diffusion resistance (R_ad_). The surge in the spectra at low frequencies indicates the large electrode polarization resistance (R_p_ = R_ct_ + R_ad_) of the silver electrodes. The resistance of O^2−^ conduction decreases slightly with an applied current of up to 20 mA, whereas there is a sharp decrease in the resistance of H^+^ conduction when the current is increased from 10 to 20 mA, indicating the influence of Joule heating. Therefore, a current within 10 mA has a slight influence on ionic conduction owing to the limited Joule heating, and a direct current of 10 mA was chosen for the conductivity tests.

### 2.3 Electrical Conductivity of BZCYYb

**Figure 3:**
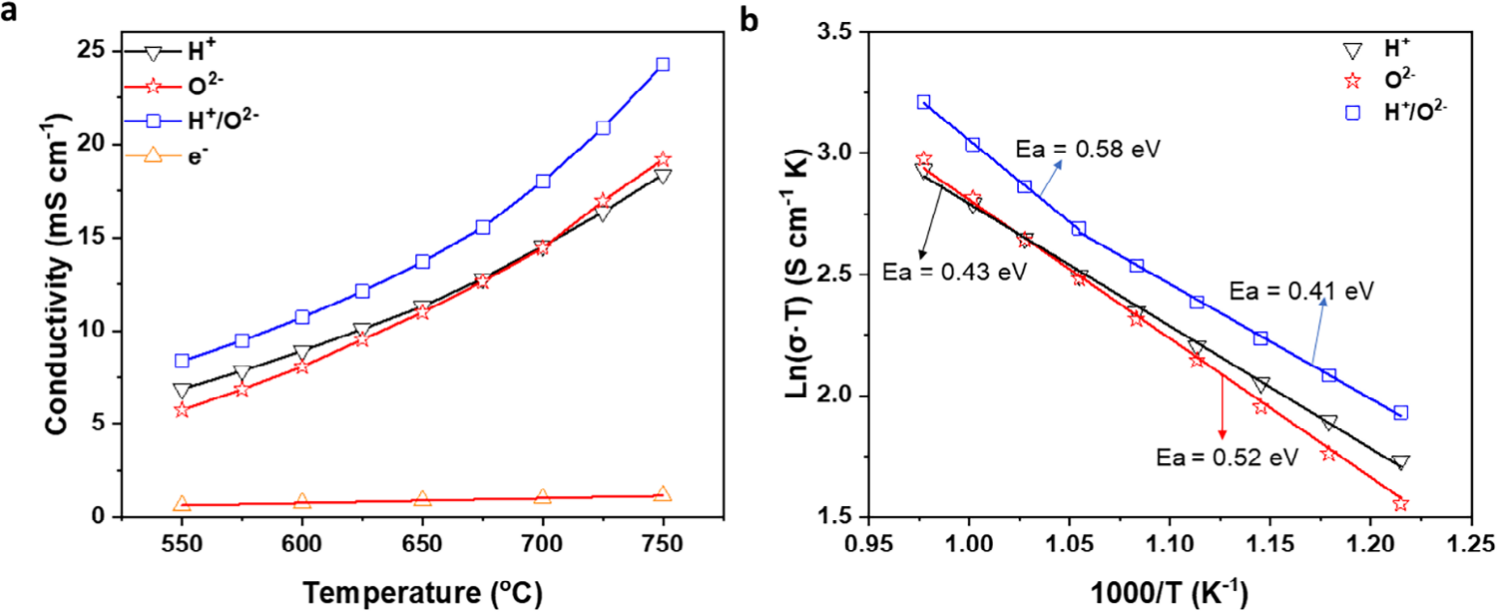
a), H^+^, O^2−^, dual‐ion and electronic conductivities of the BZCYYb membranes at different temperatures; b), Activation energies of the ionic conductivities of the BZCYYb membranes.

BZCYYb has a certain electronic conductivity,^[^ ^19^ ^]^ which cannot be excluded in the proposed method. The electronic conductivity proportionally increased from 0.63 to 1.16 mS cm^−1^ when the temperature was increased from 550 to 750 °C (Figure S4, Supporting Information). **Figure** **3** shows the electrical conductivities of the BZCYYb membranes. The ionic conductivities were tested via ESI at 10 mA, and the electronic conductivity was deducted from the ionic conductivity. Both the H^+^ and O^2−^ conductivities increase with temperature but at different rates due to the different activation energies of 0.43 and 0.52 eV for H^+^ and O^2−^ conduction, respectively. The dual‐ion conductivity is higher than the H^+^ and O^2−^ conductivities and has different activation energies at low and high temperatures owing to differences in ionic conduction. The electronic conductivity is less than 10% of the electrical conductivity. This result is consistent with the OCVs of BZCYYb‐based cells ^[^ ^19^ ^]^. Therefore, the electrical conductivity of BZCYYb is the dominant ionic conductivity.

**Figure 4:**
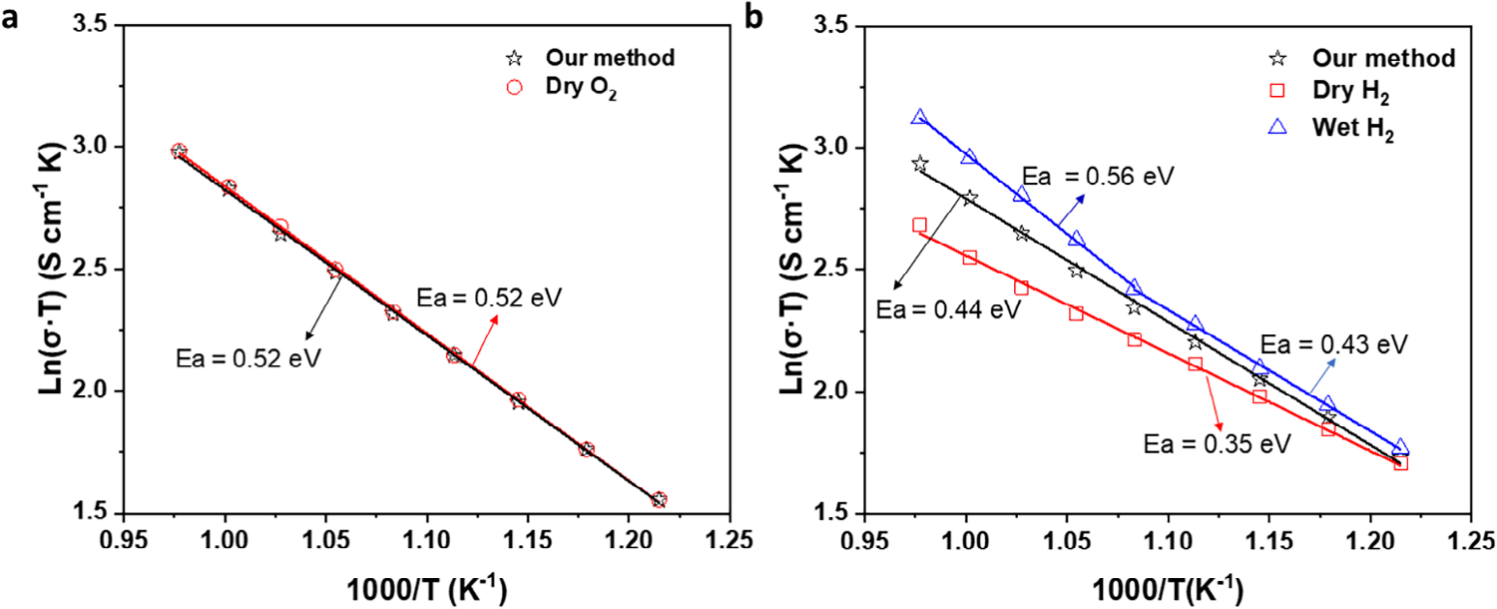
Comparison of the ionic conductivities of the BZCYYb membranes tested with the new method under a direct current of 10 mA and conventional ESI tests under OCV in different atmospheres: a), O^2−^ conductivity; b), H^+^ conductivity.

To compare the new method with conventional ESI tests, ESI tests were conducted under an OCV with the same atmosphere in both electrode chambers as conventional ESI tests. As shown in **Figure** **4**, the O^2−^ conductivity tested under the OCV in dry O_2_ is almost the same as that tested with our method, indicating that O^2−^ generated by water electrolysis can replace oxygen when testing O^2−^ conductivity (Figure S5, Supporting Information). However, the H^+^ conductivity tested in dry H_2_ is lower than that tested with our method. Different activation energies indicate that water promotes proton conduction and changes the conduction mechanism. When wet hydrogen was applied, the H^+^ conductivity tested at the OCV significantly increased, especially at high temperatures. Accordingly, water plays an important role in H^+^ conduction. Moreover, the protonic conduction in wet hydrogen demonstrated different activation energies at low and high temperatures, and the activation energy at high temperatures was higher than that at low temperatures, which suggests different conduction mechanisms within the temperature range in wet hydrogen. This is because O^2−^ conduction becomes dominant at high temperatures according to the increased activation energy, which contributes to the drastically increased conductivity. Therefore, the ionic conductivity tested under an OCV in wet hydrogen is the mixed H^+^/O^2−^ conductivity.

### 2.4 Comparison of Dual‐Ion Conductors

Dual‐ion conduction has been confirmed in several perovskite ceramics through different tests. The LSGM perovskite is generally a good O^2−^ conductor and a high H^+^ conductivity was discovered in a hydrogen atmosphere ^[^ ^14^ ^]^, which is comparable with that of BaCeO_3_‐based oxides. Taniguchi et al. reported that Gd‐doped BaCeO_3_ has lower ionic conductivity in hydrogen permeation tests than in hydrogen‒oxygen fuel cell tests owing to dual‐ion conduction,^[^ ^20^ ^]^ which was confirmed with the EMF of hydrogen and oxygen concentration cells.^[^ ^21^ ^]^ Another type of dual‐ion conductor is the composite membrane of fluorite and perovskite, such as BaZr_0.8_Y_0.2_O_3_/GDC.^[^ ^5^ ^]^ However, the dual‐ion conductivities were tested with different methods and/or under different conditions, which makes it difficult to compare their ionic conductivities. The proposed method was employed to test the individual conductivity of dual‐ion conductors under the same conditions, including LSGM, BCG, and BZCY/GDC (50:50 wt.%), which were compared with the state‐of‐the‐art BZCYYb for the first time.

**Figure 5:**
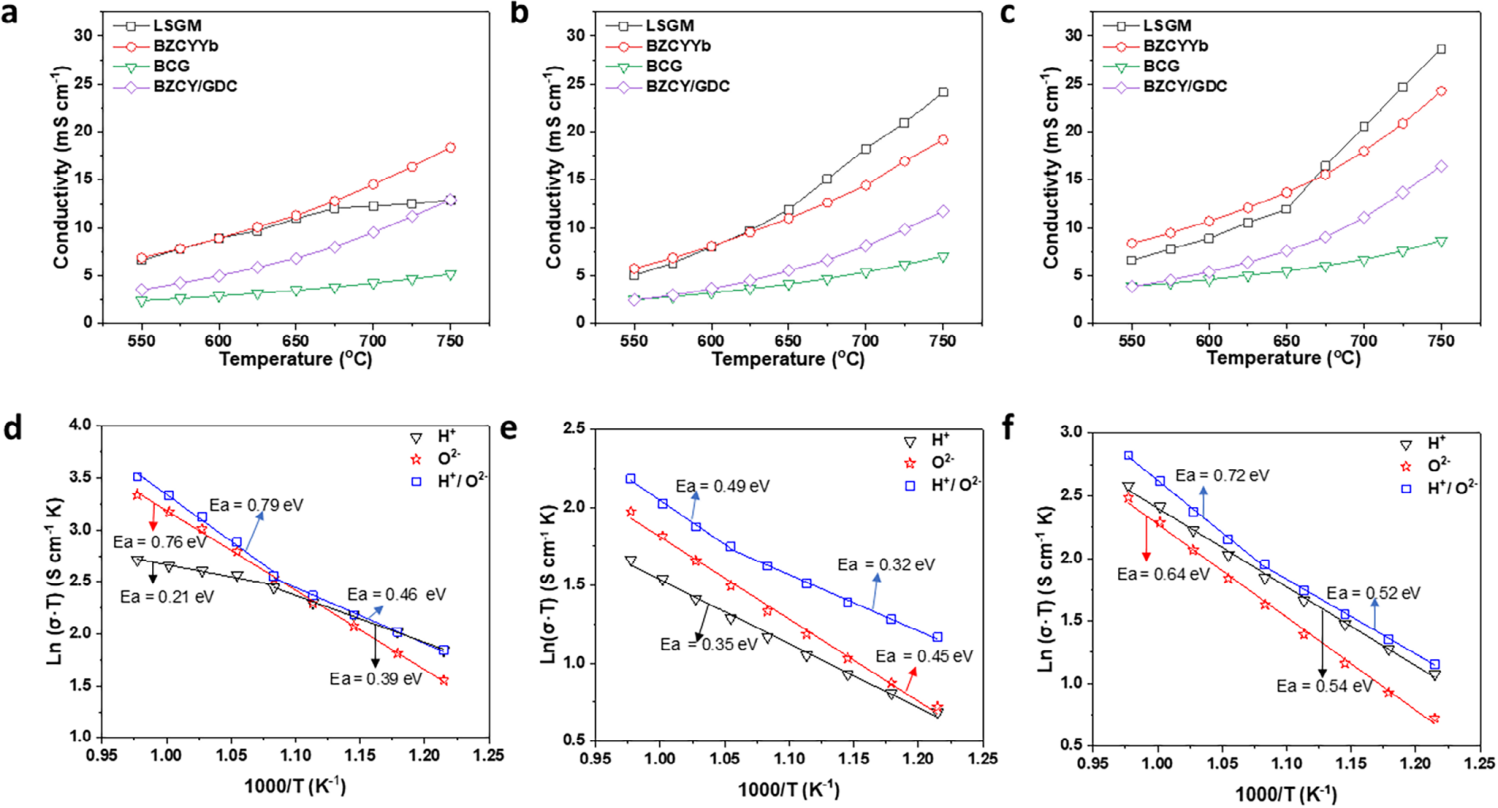
a–c), Comparison of the ionic conductivities of the BZCYYb, LSGM, BCG, and BZCY/GDC membranes: a), H^+^ conductivity; b), O^2−^ conductivity; c), dual‐ion conductivity. d–f), Arrhenius plots of the ionic conductivities of the LSGM (d), BCG (e) and BZCY/GDC (f) membranes.

As shown in **Figure** **5**, the LSGM and BZCYYb membranes have similar H^+^ and O^2−^ conductivities as well as dual‐ion conductivities from 550 to 625 °C. The H^+^ conductivity is higher than the O^2−^ conductivity in the temperature range, and both reach ≈10 mS cm^−1^ as the temperature is increased to 625 °C. At temperatures above 625 °C, the BZCYYb membrane has higher H^+^ conductivity than the LSGM membrane. According to the activation energies of ionic conduction within the temperature range (Figure 5d), a different process of proton conduction occurs at high temperatures, which makes H^+^ conductivity less dependent on temperature. This might be attributed to the increased oxygen loss at high temperatures ^[^ ^22^ ^]^. In contrast, the LSGM membrane has a much greater O^2−^ conductivity than the BZCYYb membrane at high temperatures. Accordingly, the LSGM membrane demonstrated H^+^, O^2−^ and dual‐ion conductivities comparable to those of the BZCYYb membrane at temperatures below 625 °C.

The BCG membrane has the lowest ionic conductivity, and the O^2−^ conductivity is higher than the H^+^ conductivity at temperatures ranging from 550 to 750 °C. The dual‐ion conductivity is much greater than the H^+^ and O^2−^ conductivities are, especially at low temperatures. Therefore, the BCG membrane is a dual‐ion conductor within the temperature range. The BZCY/GDC composite membranes have higher H^+^ conductivity than O^2−^ conductivity within the temperature range. The activation energy of dual‐ion conduction indicates a H^+^ conductor at temperatures below 650 °C and an O^2−^ conductor at temperatures above 650 °C. The H^+^/O^2−^ conductivity ratio can potentially be adjusted by tuning the BZCY/GDC mass ratio in the composites.

## 3 Conclusion

A simple and precise method has been developed for the measurement of the individual ionic conductivity of dual‐ion conductors. With the coupling of water electrolysis on one electrode, H^+^ and O^2−^ conductivities can be measured under a direct current, and the individual conductivities can be obtained by switching the current direction. The dual‐ion conductivity was tested, and water electrolysis occurred simultaneously on both electrodes. The partial pressure of water vapor and applied current were optimized for the measurements. With this method, the ionic conductivities of the state‐of‐the‐art BZCYYb were compared with those of other reported dual‐ion conductors, including LSGM, BCG, and BZCY/GDC. The H^+^, O^2−^ and dual‐ion conductivities of LSGM are comparable to those of BZCYYb at temperatures below 625 °C. This study provides an efficient method for measuring ionic conductivity, which has the potential to promote the development of dual‐ion conductors for various applications.

## 4 Experimental Section

### Preparation of the Ceramic Powders

BZCYYb powder was prepared via a solid‐state reaction. Stoichiometric amounts of BaCO_3_, CeO_2_, ZrO_2_, Y_2_O_3_, and Yb_2_O_3_ (Sigma Aldrich, Australia) were mixed in ethanol by ball‐milling for 24 h. The mixture was dried at 80 °C under stirring to evaporate the ethanol. The dried powder was then pressed into green pellets with a diameter of 15 mm and a thickness of ≈2 mm under a pressure of 100 MPa for 1 min via a hydraulic press, followed by sintering at 1200 °C for 10 h at a ramp rate of 2.5 °C min^−1^. Next, the pellets were ground with a mortar jar, and the obtained powder was sieved through a mesh with an aperture size of ≈8 µm.

### Characterization of the Ceramic Powders

The crystal structure of the BZCYYb powder was characterized via an X‐ray diffractometer (Miniflex 600, Rigaku, Japan) with Cu radiation. Powder X‐ray diffraction (XRD) patterns were collected in the 2θ range of 20–80° at a scanning rate of 5° min^−1^ and a step size of 0.01°.

### Membrane Preparation and Characterization

To prepare the membranes for conductivity tests, 1 wt.% NiO powder (Sigma Aldrich, Australia) was added to the BZCYYb powder as a sintering aid, and the mixture was pressed into pellets with a diameter of 20 mm and a thickness of ∼1 mm. The pellets were subsequently sintered at 1450 °C for 5 h to obtain dense BZCYYb membranes with a diameter of 15 mm. The density of the membrane was calculated as mass/volume, and the volume was measured according to Archimedes’ principle. Next, Pt paste (YURUI CHEMICAL Co., Ltd., China) was brushed on both sides of the membranes with an area of 0.63 cm^2^, followed by sintering at 1000 °C for 2 h. The microstructures of the BZCYYb membranes and Pt paste layers were observed by scanning electron microscopy (SEM, FEI, Quanta, 3D FEGSEM) at an accelerating voltage of 15 kV. The same procedure was employed for the preparation of the BCG membranes. The LSGM and BZCY/GDC membranes were prepared by pressing commercial powders (Fuelcell materials, USA) and subsequently sintering at 1450 °C for 5 h, and the mass ratio of the BZCY and GDC powders was 50%:50%.

### Ionic Conductivity Measurements

Ionic conductivities were measured via a custom‐made testing setup (Figure S1, Supporting Information). The membranes were sealed on an internal alumina tube with ceramic sealant (CAP‐552, Fuelcellmaterials, USA). Ag wires were attached to the Pt electrodes on both sides of the membranes via Ag paste. The internal tube was inserted into an external alumina tube. During the testing, argon was fed on both sides of the membranes at a flow rate of 40 sccm via the internal tube and the space between the two tubes. For the tests of H^+^ and O^2−^ conductivities, argon was passed through a water batch to carry water vapor before it intruded into the internal tube. The temperature of the water batch was controlled by a heating tape to tune the partial pressure of the water vapor in argon. For the measurement of H^+^/O^2−^ dual‐ion conductivity, argon‐carried water vapor was supplied on both sides of the membranes. EIS data were collected over membranes with an electrochemical workstation (Gamry, Interface 5000E, USA) at frequencies ranging from 100 kHz to 0.5 Hz with an AC perturbation potential of 10 mV from 550 to 750 °C. The electronic conductivity of the membranes was measured according to Ohmic law under a direct current of 10 mA.

### Statistical Analysis

All the statistical ionic conductivity calculations were conducted according to the equation

(1) $$\sigma =\frac{L}{R\cdot S}$$

where σ (S cm^−1^) represents the ion conductivity of the conductor, R (Ω) represents the ohmic resistance of the membrane according to EIS, L (cm) represents the membrane thickness, and S (cm^2^) represents the active area of the membrane.

And ohmic resistances were tested with the ESI. Each value was tested at least three times, and error bars were calculated with the standard deviation (SD) equation:

(2) $$S=\sqrt{\frac{\sum {\left(x_{i}\;-\;\mu \right)}^{2}}{N}}$$

where **S** = the population standard deviation, **N** = the size of the population, **x_i_** = each value from the population, **and μ** = the population mean.

## Conflict of Interest

The authors declare no conflict of interest.

## Supporting information



Supporting Information

## Data Availability

The data that support the findings of this study are available from the corresponding author upon reasonable request.
