# Supplementary material for: Measurement of the Proton and Oxide‐Ion Conductivities of Dual‐Ion Conductors by Switching the Current Direction
Source: Small Methods. 2025 May 19;9(8):2500166. doi: 10.1002/smtd.202500166 (PMC12391645; doi:10.1002/smtd.202500166)
Supplement: Supplementary file 1 — Supporting Information [file SMTD-9-2500166-s001.docx]

Supporting Information

Measurement of the Proton and Oxide- ion Conductivities of Dual-ion Conductors by Switching the Current Direction

Xiangcheng Liu, Qiuning Li, Lingping Zeng, Xiaoliang Zhou, Dehua Dong*, Zongping Shao*, and Huanting Wang*


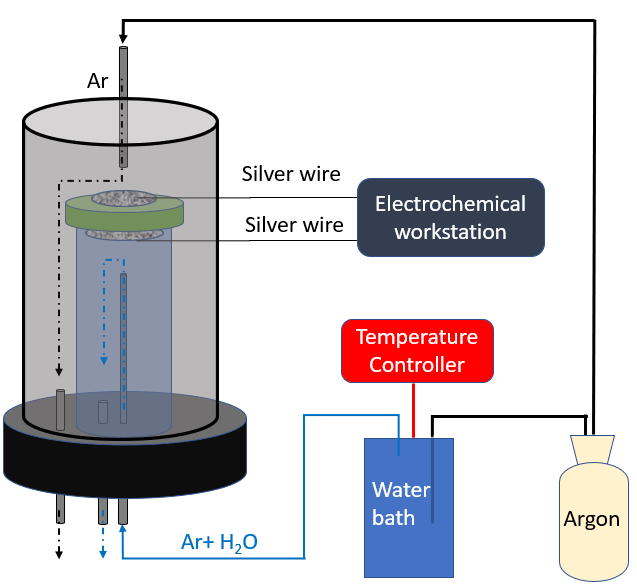


**Figure S1.** Testing setup for the tests of ionic conductivities.


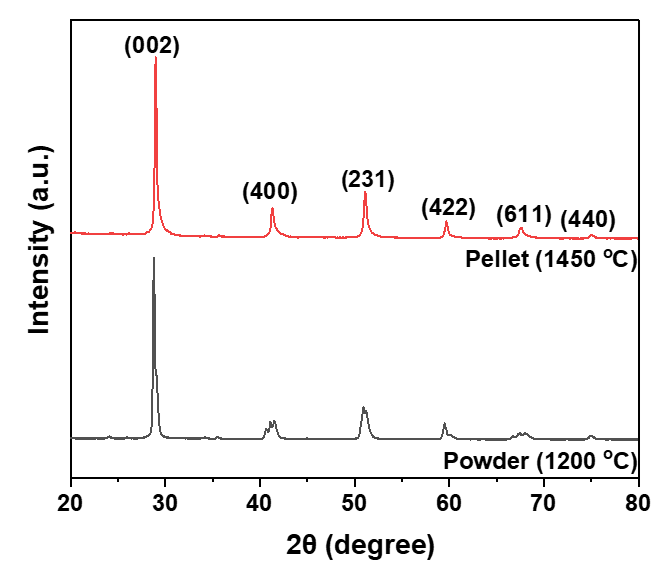


**Figure S2.** XRD patterns of BZCYYb powder and membranes.


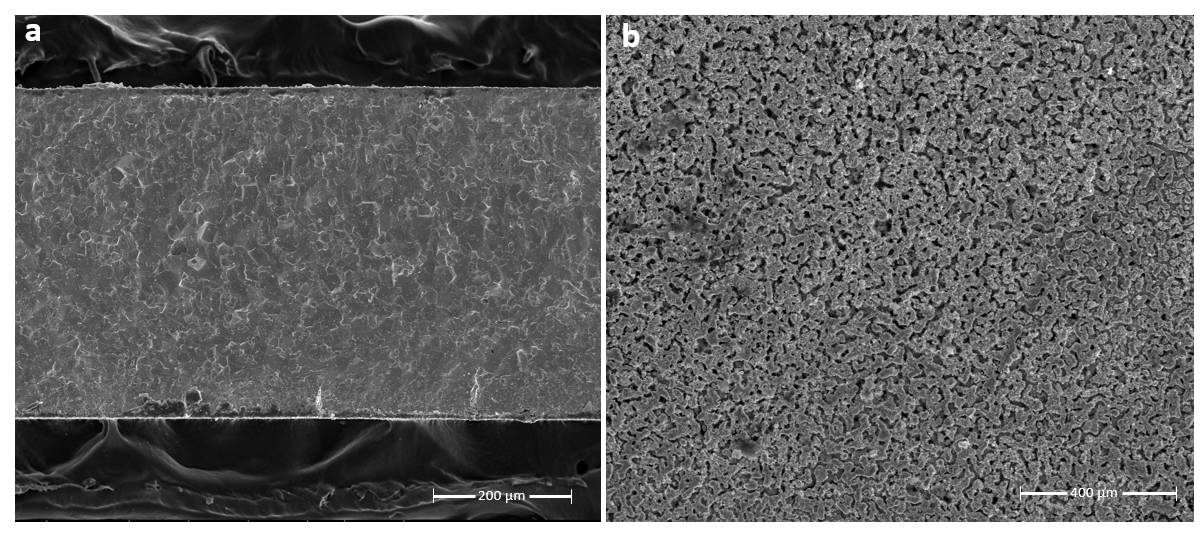


**Figure S3.** a. Cross-sectional SEM image of BZCYYb membrane; b. Surface of Pt electrodes.


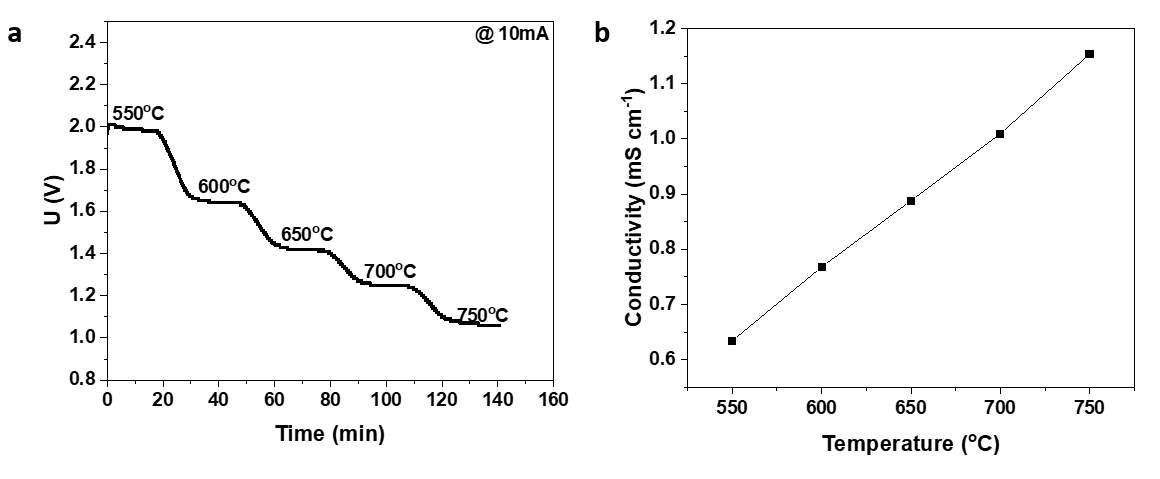


**Figure S4.** a. Voltage across BZCYYb membranes under an applied direct current of 10 mA; b. Change of electronic conductivity with temperature.


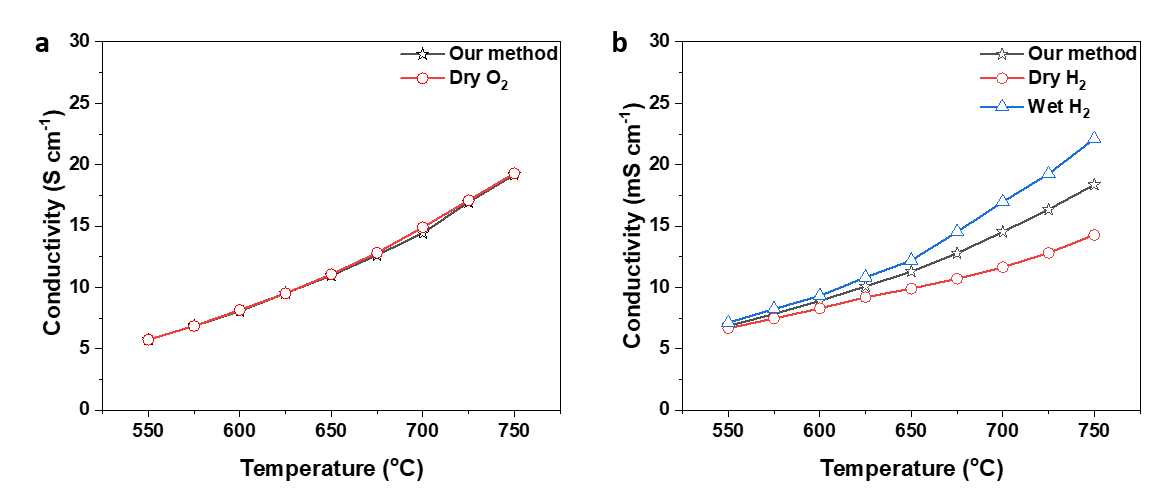


**Figure S5.** Comparison of the BZCYYb conductivities tested with the new method and conventional methods with EIS under a direct current of 10 mA and an OCV.
